# Mesenchymal Stem Cells for Cartilage Regeneration of TMJ Osteoarthritis

**DOI:** 10.1155/2017/5979741

**Published:** 2017-10-16

**Authors:** Dixin Cui, Hongyu Li, Xin Xu, Ling Ye, Xuedong Zhou, Liwei Zheng, Yachuan Zhou

**Affiliations:** ^1^State Key Laboratory of Oral Diseases & National Clinical Research Center for Oral Diseases & Department of Pediatric Dentistry, West China Hospital of Stomatology, Sichuan University, Chengdu, Sichuan 610041, China; ^2^State Key Laboratory of Oral Diseases & National Clinical Research Center for Oral Diseases & Department of Cariology and Endodontics, West China Hospital of Stomatology, Sichuan University, Chengdu, Sichuan 610041, China

## Abstract

Temporomandibular joint osteoarthritis (TMJ OA) is a degenerative disease, characterized by progressive cartilage degradation, subchondral bone remodeling, synovitis, and chronic pain. Due to the limited self-healing capacity in condylar cartilage, traditional clinical treatments have limited symptom-modifying and structure-modifying effects to restore impaired cartilage as well as other TMJ tissues. In recent years, stem cell-based therapy has raised much attention as an alternative approach towards tissue repair and regeneration. Mesenchymal stem cells (MSCs), derived from the bone marrow, synovium, and even umbilical cord, play a role as seed cells for the cartilage regeneration of TMJ OA. MSCs possess multilineage differentiation potential, including chondrogenic differentiation as well as osteogenic differentiation. In addition, the trophic modulations of MSCs exert anti-inflammatory and immunomodulatory effects under aberrant conditions. Furthermore, MSCs combined with appropriate scaffolds can form cartilaginous or even osseous compartments to repair damaged tissue and impaired function of TMJ. In this review, we will briefly discuss the pathogenesis of cartilage degeneration in TMJ OA and emphasize the potential sources of MSCs and novel approaches for the cartilage regeneration of TMJ OA, particularly focusing on the MSC-based therapy and tissue engineering.

## 1. Introduction

The temporomandibular joint (TMJ) is a hinge and gliding joint that connects the mandibular condyle with the temporal articular surface. It is one of the most frequently used joints in the human body [1]. Osteoarthritis (OA) is a group of degenerative diseases primarily affecting the joint, characterized by progressive cartilage degradation, subchondral bone remodeling, synovitis, and chronic pain [2, 3]. Osteoarthritis that happened in TMJ often involves degenerations of both hard and soft tissues of TMJ, and patients with TMJ OA usually have joint pain and dysfunction with reduced quality of life. It is estimated that approximately 15% of populations in the world suffer from OA [4]. Epidemiologic studies on the prevalence of TMJ OA differ due to variations in diagnostic criteria, and clinical evidence occurs in 8–16% of populations with symptoms of joint pain, limited occlusion motion, or TMJ sound [5]. Moreover, women have increased susceptibility to the initiation of TMJ OA and induced pain, which occurs mainly after puberty during the reproductive years [6].

The pathogenesis and underlying molecular mechanisms involved in TMJ OA development remain elusive and largely understudied [7, 8]. The treatment strategy for TMJ OA aims at preventing the progressive destruction of cartilage and the subchondral bone, relieving joint pain and restoring TMJ function. The traditional clinical treatments mainly include nonsurgical options, such as physical therapies, occlusal splints, nonsteroidal anti-inflammatory drugs (NSAIDs), and arthrocentesis [9, 10], while surgical intervention is applied to patients with severe symptoms. Although those abovementioned treatments can prevent disease progression to a certain degree, they are unable to completely restore degraded cartilage or subchondral bone lesions, as well as disc deteriorations.

Recent years, stem cell-based therapy has aroused a great attention. As a subpopulation of stem cells, mesenchymal stem cells (MSCs) have become vital seed cells for tissue regeneration due to their easy obtainment and multilineage differentiation potential. In addition, the trophic modulations of MSCs exert a biologic function in injured tissues and inflammatory diseases [11, 12]. Combined with appropriate scaffolds via transplantation *in vivo*, MSCs could restore tissue impairments to form cartilaginous or even osseous compartment in TMJ OA animal models [13, 14]. All these data indicate the capacity of MSCs for the cartilage regeneration in TMJ OA disease.

In this review, we will briefly summarize the pathogenesis of TMJ OA and emphasize the potential of novel approaches for the cartilage regeneration of TMJ OA, particularly focusing on the MSC-based therapy.

## 2. Pathogenesis of Cartilage Degeneration in TMJ OA

TMJ OA is a highly prevalent degenerative disease affecting articular cartilage as well as other TMJ tissues under pathological conditions and aging process [5]. TMJ condylar cartilage is an avascular, compressible tissue comprised of dense collagen fibres and extracellular proteoglycans, which protect the joint from damage during mechanical loading. The collagen fibres are mainly composed of type I and type II collagen (Col1/Col2) and are well organized to align in an anteroposterior orientation for the resistance to shear stress [15–17]. Chondrocytes embedded in the condylar cartilage are generally separately localized into three layers. Cells in the superficial and middle layers are considered to be progenitor cells with high proliferation capability and differentiation potential. Descending to the deep layer, chondrocytes undergo terminal differentiation with enlarged, swollen, and vacuolated morphology leading to apoptosis process. Since TMJ OA is a multifactorial disorder [18], understanding of the pathogenesis of cartilage degeneration in TMJ OA could help to identify potential therapeutic targets and interventions.

### 2.1. Chondrocyte Apoptosis

During TMJ OA progression, articular chondrocytes with low metabolism usually in advance undergo hypertrophy and apoptosis, accompanied with cartilage fibrillation and progressive loss. In cultured TMJ-derived chondrocytes, oxidative stress induced by H_2_O_2_ could elevate intracellular reactive oxygen species (ROS) and subsequently induce chondrocyte apoptosis and function impairment [19]. Intra-articular injection of monosodium iodoacetate (MIA) can effectively induce TMJ OA-like phenotype in rats and chondrocyte apoptosis as the most apparent characteristic in cartilage was observed starting from the early stage [20]. Surgical malocclusion induction in another TMJ OA rat models increased chondrocyte autophagy with a reduced activity of mitogen-activated protein kinase kinase kinase kinase-3 (MAP4K3) and mammalian target of rapamycin (mTOR) [21]. Moreover, chondrocyte apoptosis was also illustrated in the initiation stage of TMJ OA in a senescence-accelerated mouse model [22]. Collectively, all these data elucidated the involvement of chondrocyte apoptosis during the initiation and progression of TMJ OA.

### 2.2. Catabolic Enzymes

In adult healthy cartilage, chondrocytes are in a quiescent state characterized by a fine balance between anabolic and catabolic activities. However, during the disease progression, the condylar chondrocytes demonstrate the progressively decreased synthesis of anabolic components, such as Col2 and aggrecan, the upregulated expression of hypertrophic marker genes, such as Runx2 and ColX, and the increased catabolic enzymes synthesis, such as matrix metalloproteinases (MMPs) [23] and a disintegrin and metalloproteinase with thrombospondin motifs (ADAMTS) [24].

The MMPs belong to a family of proteases and function to degrade extracellular matrix proteins. Among these, MMP-13 is the most important catabolic enzymes involved in cartilage degradation during TMJ OA development. Studies have detected a higher expression of MMP-13 in late-stage OA patients compared with these in the early stage with a lower disease degree [25]. In addition, overexpression of *Mmp-13* in transgenic mice has led to degenerative cartilage with excessive Col2 cleavage and aggrecan degradation [23]. Furthermore, mice with the knockout of *Mmp-13* rescued surgically induced OA phenotype in knee joint, indicating that the cartilage damage in OA mice model is dependent on MMP-13 activity [26].

The ADAMTS family, particularly ADAMTS4 and ADAMTS5, contributes to the proteoglycan/aggrecan degradation during TMJ arthritis [27]. Inhibition of these enzymes in cultured chondrocytes effectively reduced aggrecan degradation *in vitro* [28]. The ablation of Adamts5 activity in transgenic mice prevented aggrecan loss and cartilage erosion in inflammation-induced OA models [29]. Similarly, in surgically induced OA mice models, depletion of Adamts5 or double knockout of both Adamts4 and Adamts5 protected against proteoglycan degradation *in vitro* and decreased the severity of OA progression [30]. Besides, in genetic TMJ OA mouse models, such as *β*-catenin(ex3)^Co12ER^ mice, rescue effects were also observed by deletion of the either the Mmp-13 or Adamts5 gene [31]. All these evidence suggest that the catabolic enzymes play a key role in the development of TMJ OA.

### 2.3. Subchondral Bone Remodeling

The abnormal remodeling of the subchondral bone is another pathogenic change contributed to OA in TMJ. Recent studies revealed the interaction between chondrocyte and adjacent osteoclast or osteoblast to regulate the bone-remodeling process [32]. In Camurati-Engelman disease (CED) mice with the systemic skeletal disease, the activated TGF-*β* signals in the bone marrow developed phenotypes of abnormal bone remodeling, as well as obvious cartilage degradation accompanied by an upregulation of *Mmp-9* and *Mmp-13* in condylar chondrocytes [33–35]. Besides, several lines of evidence suggested that chondrocytes can regulate bone remodeling via RANKL/osteoprotegerin (OPG) signaling. A thinner articular cartilage layer with severe destruction of growth plate cartilage was observed in OPG-deficient mice, indicating that OPG is related to the regulation of cartilage metabolism [36]. Moreover, articular chondrocytes can produce both RANKL and OPG proteins, and different RANKL/OPG ratios may adjust cartilage degradation and subchondral bone remodeling in OA [37, 38]. In malocclusion-induced TMJ OA models, the loss of subchondral bone was observed after 12 weeks, followed by increased expression of osteoclastic factors, such as M-CSF, VEGF, and RUNX, as well as the upregulated RANKL/OPG ratio [37]. To mimic OA environment *in vivo*, articular chondrocytes were cocultured with peripheral blood mononuclear cells (PBMCs) *in vitro*. Chondrocytes under the prostaglandin E2 (PGE2) stimulation paracrine secreted RANKL protein to induce osteoclastic activity of monocytes [39]. On the contrary, the *β*-catenin(ex3)^Col2CreER^ mice developed an OA-like phenotype in the knee joint characterized by subchondral bone erosion and osteophyte formation. The activation of *β*-catenin signals in chondrocytes produced OPG protein to further inhibit osteoclast differentiation by completely binding with RANK on the osteoclasts [40]. Further investigations are needed to elucidate mechanisms governing the interactions between chondrocytes and local bone remodeling; nonetheless, chondrocytes are demonstrated to serve as a link with bone changes in the OA-related process.

Apart from chondrocytes, other pathogenic factors, such as immune cells, cytokines, and hormones, lead to the pathogenic changes in the subchondral bone. Increasing evidence has demonstrated that synovium inflammation is involved in the progression of OA and associated with joint pain and dysfunction [41, 42]. Various types of immune cells are infiltrated in the inflamed synovium of OA patients. Among these, macrophages are the most abundant cells present in the synovial tissue [43, 44]. In a surgery-induced OA mouse model by intra-articular injections of collagenase into joints, synovial lining macrophages are shown to play a pivotal role in mediating the osteophyte formation during the progression of OA [45]. In cultured synovial cells derived from OA patients, synovial macrophages were specifically depleted from digested synovium using anti-CD14-conjugated magnetic beads. The macrophage depletion resulted in the downregulation of proinflammatory cytokines, such as interleukin- (IL-) 6 and IL-8 and MMPs, such as MMP-1 and MMP-3, and it indicated the important role of macrophages in promoting the production of inflammatory and degradative mediators in the OA synovium [46]. Some other studies provided clues about the inhibitory influence on the chondrogenic differentiation of MSCs under the activation of synovial macrophages [47, 48]. Despite the abundance of macrophages, natural killer cells have also been isolated from synovial tissues of OA patients; however, the mechanism involved in the pathogenesis needs to be further elucidated in detail [49].

Along with the infiltration of immune cells, inflammatory cytokines are isolated from the inflamed synovial fluid of patients with TMJ OA, such as IL-1*β* and tumor necrosis factor- (TNF-) *α* [50, 51]. In the experimental chronic inflammation of rodent TMJ induced by intra-articular injections of complete Freund's adjuvant, increased expressions of IL-1*β* and TNF-*α* were detected and supposed to be one cause for the TMJ degenerative changes [52]. Although the administration of TNF inhibitors did not show significant improvement in radiographic scores of patients, individuals have some benefits on joint pain relief and the trends suggested possible targets for the intervention of OA [53]. What is more is that several other cytokines, such as IL-6, have also been shown to be implicated in the progression of TMJ OA [51].

Females especially during the reproductive period have susceptibility to the occurrence of TMJ OA, suggesting that female hormones have a possible involvement in the pathologic changes of condylar cartilage and the subchondral bone. In an iodoacetate-induced TMJ OA rat model, estrogen can aggravate the disease progression by upregulating Fas- and caspase 3-related proapoptotic genes [54]. On the contrary, estrogen could inhibit the expression of nitric oxide to protect TMJ chondrocyte from apoptosis [55]. Thus, the role of estrogen in TMJ OA progression is with controversy and needed more investigations.

## 3. Mesenchymal Stem Cell-Based Therapy for Cartilage Regeneration of TMJ OA

The treatment for TMJ OA focuses on preventing the destruction of cartilage and subchondral bone, relieving pain, and eventually restoring TMJ function. There are different treatment strategies according to clinical stages of TMJ degeneration, including noninvasive options, such as physical therapies, occlusal splints, NSAIDs, arthrocentesis [9], and surgical intervention, such as joint replacement [56]. Although all these therapies have symptom-modifying and/or structure-modifying effects to some extent, they rarely reverse the disease process to restore the degenerative cartilage and reestablish joint functions.

In recent years, stem cells have been extensively applied to fields of tissue engineering and regenerative medicine [57–59], mainly due to their self-renewal ability and multiple differentiation potentials. Among alternative cell sources for OA treatment, MSCs have raised particular concerns to play a role of seed cells, based on their ease of collection, the potential of chondrogenic differentiation, response to tissue damage, and contributions to tissue turnover. Therefore, extensive progress has been made in the investigation of differentiation potentials and functional modulations of MSCs for cartilage regeneration in TMJ OA treatment.

### 3.1. Potential of Mesenchymal Stem Cells

MSCs have been identified from various tissues, such as skeletal muscle [60], adipose tissue [61], the placenta [62], the bone [63], the deciduous teeth [64], and the synovium [65]. As the most widely studied sources of MSCs for cartilage regeneration, bone marrow-derived MSCs, synovium-derived MSCs, and umbilical cord-derived MSCs are mainly discussed below.

#### 3.1.1. Bone Marrow-Derived MSCs

MSCs were first identified in the bone marrow via the formation of colonies, represented as colony-forming unit fibroblasts (CFU-F) [66], and bone marrow-derived MSCs (BMSCs) have multilineage differentiation potentials [63], including chondrogenic and osteogenic differentiation.

Studies found that cultured BMSCs preconditioned in osteogenic and chondrogenic media *in vitro* can form bone-like and cartilage-like structures, respectively, mimicking a primordial joint-like structure when seeded in opposite portions of a hyperhydrated collagen gel via ultrarapid tissue engineering techniques [67]. Some clinical trials have proposed the approaches for OA treatment, which involve the intra-articular injection to deliver BMSCs directly into the synovial fluid compartment [68]. Although most clinical trials participated in the intervention of OA in knee joints, studies on the cartilage regeneration of BMSCs in TMJ OA have been largely investigated. Chen et al. [69] conducted intra-articular injections of both undifferentiated and prechondrogenic differentiated BMSCs for cartilage regeneration in TMJ OA rabbit models. They also compared that the differentiated MSC-treated group gained better histological scores than the undifferentiated MSC-treated group at an observation period of 4 and 12 weeks, along with decreased expression of MMP-13 and upregulation of Sox9, Col2, and aggrecan. However, the rescue effect displayed no difference in both groups until 24 weeks. Collectively, local delivery of chondrogenic differentiated BMSCs may enhance the regenerative process of cartilage repair at the early stage of TMJ OA through key mediators involved in chondrogenesis. In addition, the implanted cells could be traced by the label of adenoviral vectors containing the LacZ gene, and implanted MSCs were detected within cartilage, subchondral bone, and synovium lasting at least 4 weeks, indicating the involvement of BMSCs in the cartilage repair [69].

Although predifferentiated BMSCs appear to enhance the cartilage regeneration, they do not maintain their proliferative capability and differentiation potentials after prolonged expansion *in vitro* [70]. In order to attain a better therapeutic outcome, applicable strategies of pretreatment and/or preconditioning of BMSCs are essential to improve long-term effect in OA. Further studies have proved that pretreatment of BMSCs with fibroblast growth factor-2 (FGF-2) [71] and hypoxic preconditioning of BMSCs [72] are two attractive approaches to enhance cell proliferation and chondrogenic differentiation. Whether there are other biophysical approaches needs more investigations to expand the applications of BMSCs in cell-based treatment. Therefore, BMSCs provide an alternative approach for cartilage regeneration; meanwhile, a better application of TMJ OA treatment could be achieved with the pretreatment of BMSCs.

#### 3.1.2. Synovium-Derived MSCs

A number of studies have isolated cells from synovial fluid and synovium in TMJ [73–75], which are able to differentiate into different lineages, such as osteoblasts, chondrocytes, adipocytes, and neurons [73, 76]. These synovium-derived MSCs express MSC markers such as CD90, CD105, and CD73 and negatively express CD11b, CD19, CD34, CD45, and HLA-DR [75], compliant with the widely adopted criteria stipulated by the International Society for Cellular Therapy (ISCT) [77]. Compared with MSCs from other tissues, synovium-derived MSCs possess a greater proliferative rate and superior chondrogenic differentiation potential [78–80]. Particularly, the combination treatment of TGF-*β*s, dexamethasone, and BMP-2 became the optimum for chondrogenic differentiation of synovium-derived MSCs *in vitro* [81]. Based on the properties mentioned above, synovium-derived MSCs have been utilized to test the chondrogenic potential for cartilage repair in animal models. In rabbit OA models with defects in whole cartilage layers, synovium-derived MSCs were embedded in collagen gel and transplanted into the injury site. The cartilage defects were repaired with productions of cartilage matrix [82]. The *in vivo* chondrogenic differentiation of synovium-derived MSCs on cartilage repair have been reported in many other studies [83, 84], leading to a general acceptance that synovium-derived MSCs have the ability to repair cartilage defects to some extent.

The perforation of TMJ disc tissue is usually happening in the late stage of TMJ OA, leading to severe degeneration of condylar cartilage. The application of synovium-derived MSCs for the TMJ disc repair has aroused great attention. A recent study [85] has cultured synovium-derived MSCs on fibrin/chitosan hybrid scaffold under chondrogenic induction combined with TGF-*β*3 *in vitro.* In order to evaluate the *in vivo* repair ability, the construct was inserted into the punched TMJ disc explants of rats, which can mimic TMJ disc perforation in human. After 4 weeks of operation, distinct fibrocartilage formation with deposition of Col1 and Col2 was observed at the implantation site. Thus, synovium-derived MSCs are able to repair the defective cartilage in TMJ disc.

#### 3.1.3. Umbilical Cord-Derived MSCs

Apart from adult tissue-derived MSCs, MSCs could also be isolated from the umbilical cord (UC) [86, 87]. Compared with BMSCs, UC-derived MSCs show a more similar gene expression profile to that of embryonic stem cells [88]. They possess a faster proliferative rate and a larger number of CFU-F [89, 90].^.^The differentiation capacity of UC-derived MSCs into adipogenic, chondrogenic, and osteogenic lineages has been extensively studied [87, 90]. For cartilage tissue engineering, UC-derived MSCs and BMSCs embedded in the polyglycolic acid (PGA) scaffolds and cultured under chondrogenic differentiation medium *in vitro*. After 3 to 6 weeks, UC-derived MSCs produced more glycosaminoglycans (GAGs) and Col1 than BMSCs, indicating the superior capability of fibrochondrogenesis of UC-derived MSCs [91]. Above all, UC-derived MSCs might be an alternative cell source for the cartilage regeneration.

### 3.2. Trophic Modulations of MSCs for Cartilage Tissue Regeneration of TMJ

It is well known that MSCs are able to secrete a broad range of bioactive molecules, such as growth factors, cytokines, and chemokines, which constitutes their biological role under injury conditions [92–94]. These trophic factors produced in the MSC-conditioned medium are collectively described as the MSC secretome. Various MSC-based clinical trials have revealed that transplanted MSCs exert biological functions through trophic modulations rather than differentiation potential [11, 12]. This paradigm shift in the use of MSC-based therapy is becoming a hot issue attracting various studies for better applications to tissue regeneration.

Although TMJ OA is classified as a “low-grade-inflammatory arthritic condition” [9], the involvement of inflammation has been concerned to play a role in disease progression. Several inflammatory cytokines are increased in the synovial fluid of TMJ OA patients, such as IL-12, IL-1*β*, and TNF-*α* [51]. Also, the increased expressions of IL-1*β* and TNF-*α* were detected in rat TMJ OA models. Particularly, the biomechanical properties of TMJ discs were significantly decreased and the disc ultrastructures were impaired in rodent TMJ OA [52], implying that the chronic inflammation in TMJ OA deteriorates the adaptive ability of the joint. Since MSCs have been explored to regenerate damaged tissue and treat inflammation in many diseases, such as cardiovascular disease, neuron injury, stroke, diabetes, and bone regeneration [95, 96], further studies elucidated that the trophic factors secreted by MSCs exert an anti-inflammatory effect. van Buul et al. added TNF-*α* and interferon-*γ* (IFN-*γ*) to conditioned medium of MSCs to mimic the inflammatory environment of OA. It was found that the increased secretion of secretome decreased expression of the inflammatory gene *IL-1β* and collagenase genes *Mmp-1* and *Mmp-13* in response to inflammation [97]. Besides, human BMSCs were intra-articularly injected into rat's knee joint after hemimeniscectomy [97]. Despite the rapid decrease of cell numbers, the human BMSC injection enhanced Col2 expression in the articular cartilage, associated with increased expression of Indian hedgehog (Ihh), parathyroid hormone-like hormone (PTHLH), and bone morphogenetic protein 2 (BMP2), eventually contributing to the cartilage regeneration and inhibition of OA progression [98]. Furthermore, the periodontal ligament-derived MSCs could increase the cell proliferation and matrix biosynthesis of cocultured TMJ-derived fibrochondrocytes through paracrine secretion of trophic factors [99]. In addition, increased GAG deposition with enhanced expression of chondrogenic genes, such as aggrecan, Col1, and Col2, is also observed in this study.

As a component of secretome, exosomes are demonstrated to play a key role in mediating tissue repair in MSC-based therapy. Exosomes are cell-secreted nano-sized vesicles covered by the bilipid membrane and containing a myriad of regulatory components including microRNAs (miRNAs), mRNAs, and proteins [100–102]. Exosomes can be synthesized in many cells, such as lymphocytes, dendritic cells, and tumor cells, and they are found in most bodily fluids such as blood, urine, and saliva [103]. Diseased cells also secret exosomes as vehicles to transmit injurious signals, thereby exerting various pathological effects on both recipient and parent cells [104–106]. In MSC-based regenerative therapy, exosomes are widely found in the secretome of MSCs derived from the bone marrow [107, 108], fetal tissues [109], and umbilical cord [110]. To isolate exosomes from secretomes, the conventional culture medium is replaced by medium containing exosome-depleted fetal bovine serum. When BMSCs reach 60–80% confluence, cell culture media are collected and density gradient centrifugations are performed to obtain pellets containing exosomes. The pellets are then passed through a 0.22 *μ*m filter to remove cell debris, and the purified exosomes are obtained [108].

Although the role of the individual components of exosome has not been elucidated, the combined functional complexity of MSC exosomes has therapeutic effects on tissue repair and regeneration in the heart [100], liver [111], skin [112], bone [113], and cartilage [114]. In an experimental rat model with critical-sized osteochondral defects on trochlear grooves of the distal femurs, human embryonic MSC-derived exosomes were intra-articularly administrated to investigate the efficacy of exosomes in osteochondral repair. The results showed that over the 12-week period of exosome injections, exosome-treated cartilage and subchondral bone defects were completely restored, characterized by the accelerated neotissue filling and enhanced matrix synthesis of Col2 and sulphated GAG, while only fibrous repair tissues were found in the PBS-treated defects [114]. A recent study conducted the destabilization of medial meniscus (DMM) surgery to induce OA in the knee joints of mice, and the intra-articular injections of exosomes isolated from embryonic MSC medium successfully impeded cartilage destruction in the DMM model. Further *in vitro* studies using cultured chondrocytes treated with IL-1*β* illustrated that these exosomes maintained the chondrocyte phenotype by increasing Col2 synthesis and decreasing ADAMTS5 expression, exerting a beneficial therapeutic effect on OA through balancing synthesis and degradation of cartilage extracellular matrix [115]. Among components in the exosome, miRNA might participate in mediating the efficacy of MSC exosome against OA. Exosomal miR-23b could inhibit protein kinase A (PKA) signaling to induce chondrogenic differentiation of human MSCs [116]. Moreover, overexpression of miR-125b in human OA chondrocytes can suppress the IL-1*β*-induced upregulation of ADAMTS4, and in silico analysis further predicted ADAMTS4 as a putative target gene of miR-125b can be directly regulated in chondrocytes during OA development [117].

All these results demonstrated that the trophic modulations of MSCs play an essential role in the cartilage regeneration in TMJ OA. However, more investigations are needed to figure out the exact effective components among various trophic factors produced by MSCs, which could not only fulfill the current understanding of MSC-based therapy under abberant environment but also enable allogenic transplantations in a more controlled manner for better applications of tissue regeneration.

### 3.3. Tissue Engineering Approaches for Cartilage Tissue of TMJ

Based on the rapid development of scaffold materials, the cartilage regeneration of TMJ has been extensively explored in tissue engineering investigations [118–120].

Polyglycolic acid (PGA) is one of the widely applied biocompatible materials in tissue engineering of TMJ cartilage. Studies have proved that PGA enables proliferation of porcine TMJ disc cells, and matrix deposition of Col1 was detected in PGA nonwoven meshes [121, 122]. However, the rapid degradation of PGA is the major demerit leading to the loss of structural/mechanical integrity and construct contraction over time. Due to the defects of PGA, polylactic acid (PLA) has emerged as a more applicable biomaterial for TMJ cartilage regeneration. Porcine TMJ disc cells seeded in PLA had the increased cellularity, with more matrix deposition of collagen and GAGs compared with these in PGA construct [123]. Specifically, adverse construct contraction was not observed in PLA constructs compared to PGA. Another study developed a PLA construct with autologous adipose-derived MSCs predifferentiated in chondrogenic medium. After transplantation in rabbit TMJ, a regular and calcified surface of condyle cartilage was observed, accompanied by the increased expression of Col2 [124].

Except for these scaffolds, syntheses with synthetic polymers, such as PGA and PLA, natural biopolymers, such as fibrin and chitosan, are also applied to the TMJ cartilage regeneration based on their inherent advantage of biocompatibility. A recent study showed that fibrin could improve cell seeding, proliferation, and chondrogenic differentiation *in vitro* [85]. Moreover, a combination of fibrin/chitosan scaffolds can promote the reparative ability of synovium-derived MSCs, characterized by the fibrocartilage formation with extracellular matrix deposition of Col1, Col2, and GAGs in rat models with TMJ disc explants [85].

In addition to those preceding biopolymers, biologic scaffold materials consisting of extracellular matrix (ECM), such as decellularized urinary bladder matrix (UBM), have aroused great attention in the field of cartilage regeneration. These biologic scaffolds possess constructive remodeling properties by promoting the de novo formation of site-appropriate and functional host tissues, and they are already applied to various preclinical studies and clinical trials [125, 126]. In a canine model of TMJ discectomy, decellularized UBM scaffold acted as an effective interpositional material for the TMJ disc remodeling with no secondary pathologic changes. Of note, the UBM device was further replaced by native tissues, including fibrocartilage, muscle, and connective tissues. This remodeled device consists of elongated fibroblast-like cells within a highly aligned matrix of collagen and site-appropriate soft tissue attachments at the periphery of the implanted material [127].

Considering the pathological features of TMJ OA, emerging investigations have emphasized the pretreatments on the scaffold to construct the stratification of cartilage and the subchondral bone from a single source of MSCs [128]. BMSCs were induced into chondrogenic and osteogenic lineages, and then encapsulated in the stratified polyethylene glycol- (PEG-) based hydrogels to further photopolymerize into human-shaped condyle. After transplantation *in vivo*, distinct cartilaginous and osseous compartments of the mandibular condyle were formed, with the expression of cartilage-related Col2 and GAGs in the chondral layer and osteogenic markers alkaline phosphatase and osteonectin in the osteogenic layer [13].

Moreover, injectable biomaterial scaffolds have been designed to act as delivery systems containing both cells and biomolecules for more effectively modulating stem cell fate and functions [129, 130]. It has the potential to sustain stem cell survival and signal release [131]. The injectable scaffolds with a lower crosslinked degree and matrix stiffness can promote the chondrogenic differentiation of encapsulated MSCs and increase the matrix biosynthesis of Col2 and GAGs [132]. Furthermore, changes on the mechanical parameters of scaffolds could modulate the MSC differentiation to form different cartilage tissues. MSCs in scaffolds of higher cross-linking degree tend to differentiate into fibroblasts and subsequently form fibrous/osteochondral tissues in the OA rabbit model [133]. Collectively, the scaffold architecture can influence the lineage differentiation of MSCs and the mechanical parameters should be fully estimated to improve MSC-based therapy and tissue engineering.

In summary, the ideal scaffold could incorporate specific biomolecules and growth factors and enhance both chondrogenic and osteogenic differentiation potential of MSCs under different external stimuli, thus providing better applications in cartilage regeneration in TMJ OA via tissue engineering.

## 4. Conclusion

Given the limited self-healing potentials of avascular cartilage, little effective therapy is available for the repair of normal TMJ tissues in OA disease. Although the conventional nonsurgical or surgical treatments can relieve the joint pain to some extent, they cannot completely restore the TMJ function and reverse disease progression. MSCs, which have the multilineage differentiation potentials, may provide an alternative treatment for the cartilage degradation in TMJ OA. Combined with the trophic modulations of MSCs and various scaffold application, the formation of cartilaginous compartment or even stratified cartilaginous and osseous compartments has been accomplished in TMJ OA animal models. Furthermore, continuous investigations are required to detect the target efficiency and biocompatibility in the therapeutic intervention of TMJ OA, hopefully towards the preclinical and clinical researches like in OA treatment.
